# Acknowledgement to Reviewers of *Biology* in 2014

**DOI:** 10.3390/biology4010039

**Published:** 2015-01-08

**Authors:** Biology Editorial Office

**Affiliations:** MDPI AG, Klybeckstrasse 64, CH-4057 Basel, Switzerland

The editors of *Biology* would like to express their sincere gratitude to the following reviewers for assessing manuscripts in 2014:

Angata, Takashi
Ann, David K.
Arany, Istvan
Arenz, B. E.
Autexier, Chantal
Axer, Hubertus
Barres, Romain
Basenko, Evelina
Behrens, Sarah
Bell, Christopher G.
Beutler, John A.
Bonati, Laura
Bonvin, Alexandre
Branco, Miguel
Brissette, Catherine A.
Burris, Heather H
Cairns, David
Caminiti, Giuseppe
Chen, Yi-Ting
Cohick, Wendie
Coll, Julio
Connell, Laurie
Cooper, Helen J.
Cooper, Robin
Dacks, Joel B.
Dave, Kunjan R.
Davidson, Michael H.
Davis, Aaron M
Debold, Ned
Dilworth, F. Jeffrey
Dramsi, Shaynoor
Drummer, Heidi
Du, Yanan
Dufau, Maria L.
Dunlap, Jay
Eggertsen, Gosta
Facchiano, Angelo
Ferbeyre, Gerardo
Fernandez, Maria Luz
Finak, Greg
Flisikowski, Krzysztof
Foret, Frantisek
Fowler, Velia M.
Fox, Jennifer L.
Francis, Austin
Gallicano, G. Ian
Gay, Denise L.
Gayan-Ramirez, Ghislaine
Gestwicki, Jason E.
Ginsberg, Henry N.
Gissi, Carmela
Gist, Nicholas
Glazier, Douglas S.
Grafton, Gillian
Gray, Jack
Gunseli, Eren
Guyot, Romain
Harris, Marc
Hinz, Boris
Hiroaki, Hidekazu
Ichiyanagi, Tomoko
Ikawa, Yoshiya
Ilkka, Heinonen
Ishii, Tomohiro
Ivarsson, Ylva
Jameson, David
Jaudszus, Anke
Jeffery, Constance J.
Júnior, Edward Araujo
Kelley, Lawrence
Kerfoot, James
Khurana, Tejvir S.
Kim, Y.O.
Kitamoto, Toshi
Kitson, Alex
Kizilirmak, Jasmin M.
Kley, Rudolf Andre
Knip, Danielle M.
Kong, Siu-Kai
Kubota, Sho
Kuwahara, Masayasu
Lee, Hong Kyu
Lee, Hsinyu
Lenaerts, C.
Li, Yujing
Linhart, Heinz
Loizides, Ursula
Macas, Jiří
Maccani, Matthew
Marques, Eliza
Martin, William
Mavros, Yorgi
McLean, Brendan
Midzak, Andrew
Miller, Mark
Miyata, Naoyuki
Miyoshi, Eiji
Murphy, Therese
Nadaj-Pakleza, A. A.
Nagaraj, Nagarhndsm
Nees, Matthias
Nelson, William G
Neubig, Richard R.
Ohlendieck, Kay
Pan, Sheng
Panagiotidis, Mihalis
Paradiso, Angelo
Pauli, Beatriz Diaz
Pavlova, Elena
Phillips, Michael
Pico, Alex
Pinney, Sara
Piras, Paolo
Pirro, Valentina
Preuss, Mary
Quinlivan, Rosaline
Rayfield, Emily J.
Reis, Victor
Rodino-Klapac, Louise R.
Rouault, Tracey A.
Rowe, Glenn
Ruehl, Ralph
Russell, Mark W.
Ryan R. Brinkman, Ryan R.
Sacco, Margherita
Sánchez, Borja
Schebb, Nils Helge
Schulz, Benjamin L.
Shannon, Paul
Shen, Jian
Shindou, Hideo
Singh, Chandra K.
Smart, Neil
Smith, Jonathan
Strasser, Barbara
Taly, Antoine
Tamura, Koji
Tani, Tokio
Tehlivets, Oksana
Theda, Christiane
Thompson, Loren P.
Tokumura, Akira
Tomlinson, Sean
Tosi, Luiz
Trinick, John
Uchiyama, Susumu
Vaiman, Daniel
Valavanidis, Athanasios
VanNess, J. Mark
Vermeulen, Michiel
Vinknes, K.J.
Waisenegger, Michael
Wakabayshi, Katsuzo
Walsh, Kenneth
Watson, Keith G
Weiss, Robert S.
Wenderoth, Nicole
Wilson, Melinda E.
Winge, Dennis R.
Wong, Nicholas C.
Wu, Jun
Wu, Meng
Yan, Y. Sanders
Young, G.Bryan
Youngson, Neil
Zacharias, Martin
Zeisberg, Michael
Zhang, Yan

